# Supplementary material for: Resolving the full spectrum of human genome variation using Linked-Reads
Source: Genome Res. 2019 Apr;29(4):635–45. doi: 10.1101/gr.234443.118 (PMC6442396; doi:10.1101/gr.234443.118)
Supplement: Supplemental Material [file supp_gr.234443.118_Supplemental_longranger-2.2.zip › longranger-2.2.tar.bz2!/lr2.2-public/lib/stan/cmdstan/stan/doc/api/html/lbfgs__update_8hpp_source.html]

Stan: src/stan/optimization/lbfgs\_update.hpp Source File


|  |  |
| --- | --- |
|  | Stan  2.5.0  probability, sampling & optimization |


- Main Page
- Related Pages
- Namespaces
- Classes
- Files

- File List
- File Members

All Classes Namespaces Files Functions Variables Typedefs Enumerations Enumerator Friends Macros Pages

- src
- stan
- optimization

lbfgs\_update.hpp

Go to the documentation of this file.

1 #ifndef STAN\_\_OPTIMIZATION\_\_LBFGS\_UPDATE\_HPP

2 #define STAN\_\_OPTIMIZATION\_\_LBFGS\_UPDATE\_HPP

3

4 #include <vector>

5

6 #include <boost/tuple/tuple.hpp>

7 #include <boost/circular\_buffer.hpp>

8

9 #include <stan/math/matrix/Eigen.hpp>

10

11 namespace stan {

12  namespace optimization {

18  template<typename Scalar = double,

19  int DimAtCompile = Eigen::Dynamic>

20  class LBFGSUpdate {

21  public:

22  typedef Eigen::Matrix<Scalar,DimAtCompile,1> VectorT;

23  typedef Eigen::Matrix<Scalar,DimAtCompile,DimAtCompile> HessianT;

24  typedef boost::tuple<Scalar,VectorT,VectorT> UpdateT;

25

26  LBFGSUpdate(size\_t L = 5) : \_buf(L) {}

27

33  void set\_history\_size(size\_t L) {

34  \_buf.rset\_capacity(L);

35  }

36

48  inline Scalar update(const VectorT &yk, const VectorT &sk,

49  bool reset = false) {

50  Scalar skyk = yk.dot(sk);

51

52  Scalar B0fact;

53  if (reset) {

54  B0fact = yk.squaredNorm()/skyk;

55  \_buf.clear();

56  }

57  else {

58  B0fact = 1.0;

59  }

60

61  // New updates are pushed to the "back" of the circular buffer

62  Scalar invskyk = 1.0/skyk;

63  \_gammak = skyk/yk.squaredNorm();

64  \_buf.push\_back();

65  \_buf.back() = boost::tie(invskyk,yk,sk);

66

67  return B0fact;

68  }

69

78  inline void search\_direction(VectorT &pk, const VectorT &gk) const {

79  std::vector<Scalar> alphas(\_buf.size());

80  typename boost::circular\_buffer<UpdateT>::const\_reverse\_iterator buf\_rit;

81  typename boost::circular\_buffer<UpdateT>::const\_iterator buf\_it;

82  typename std::vector<Scalar>::const\_iterator alpha\_it;

83  typename std::vector<Scalar>::reverse\_iterator alpha\_rit;

84

85  pk.noalias() = -gk;

86  for (buf\_rit = \_buf.rbegin(), alpha\_rit = alphas.rbegin();

87  buf\_rit != \_buf.rend();

88  buf\_rit++, alpha\_rit++) {

89  Scalar alpha;

90  const Scalar &rhoi(boost::get<0>(\*buf\_rit));

91  const VectorT &yi(boost::get<1>(\*buf\_rit));

92  const VectorT &si(boost::get<2>(\*buf\_rit));

93

94  alpha = rhoi \* si.dot(pk);

95  pk -= alpha \* yi;

96  \*alpha\_rit = alpha;

97  }

98  pk \*= \_gammak;

99  for (buf\_it = \_buf.begin(), alpha\_it = alphas.begin();

100  buf\_it != \_buf.end();

101  buf\_it++, alpha\_it++) {

102  Scalar beta;

103  const Scalar &rhoi(boost::get<0>(\*buf\_it));

104  const VectorT &yi(boost::get<1>(\*buf\_it));

105  const VectorT &si(boost::get<2>(\*buf\_it));

106

107  beta = rhoi\*yi.dot(pk);

108  pk += (\*alpha\_it - beta)\*si;

109  }

110  }

111

112  protected:

113  boost::circular\_buffer<UpdateT> \_buf;

114  Scalar \_gammak;

115  };

116  }

117 }

118

119 #endif

stan::optimization::LBFGSUpdate::search\_direction

void search\_direction(VectorT &pk, const VectorT &gk) const

Compute the search direction based on the current (inverse) Hessian approximation and given gradient...

**Definition:** lbfgs\_update.hpp:78

stan::optimization::LBFGSUpdate::HessianT

Eigen::Matrix< Scalar, DimAtCompile, DimAtCompile > HessianT

**Definition:** lbfgs\_update.hpp:23

stan::optimization::LBFGSUpdate::update

Scalar update(const VectorT &yk, const VectorT &sk, bool reset=false)

Add a new set of update vectors to the history.

**Definition:** lbfgs\_update.hpp:48

stan::optimization::LBFGSUpdate::LBFGSUpdate

LBFGSUpdate(size\_t L=5)

**Definition:** lbfgs\_update.hpp:26

stan::optimization::LBFGSUpdate::\_buf

boost::circular\_buffer< UpdateT > \_buf

**Definition:** lbfgs\_update.hpp:113

stan::optimization::LBFGSUpdate::set\_history\_size

void set\_history\_size(size\_t L)

Set the number of inverse Hessian updates to keep.

**Definition:** lbfgs\_update.hpp:33

stan::optimization::LBFGSUpdate::UpdateT

boost::tuple< Scalar, VectorT, VectorT > UpdateT

**Definition:** lbfgs\_update.hpp:24

stan::optimization::LBFGSUpdate::VectorT

Eigen::Matrix< Scalar, DimAtCompile, 1 > VectorT

**Definition:** lbfgs\_update.hpp:22

Eigen.hpp

stan::optimization::LBFGSUpdate::\_gammak

Scalar \_gammak

**Definition:** lbfgs\_update.hpp:114

stan::optimization::LBFGSUpdate

Implement a limited memory version of the BFGS update.

**Definition:** lbfgs\_update.hpp:20


---

[ Stan Home Page ]

*© 2011–2014,
Stan Development Team.*
